# Plasticity of HIV-specific CD8 T cell responses in untreated HIV-1 infection- a step towards a therapeutic vaccine against drug resistance mutations

**DOI:** 10.1186/1742-4690-9-S2-P284

**Published:** 2012-09-13

**Authors:** J Roider, A Kalteis, T Vollbrecht, L Gloning, R Stirner, N Henrich, JR Bogner, R Draenert

**Affiliations:** 1 University of Munich, Munich, Germany

## Background

For a therapeutic HIV-1 vaccine, it should be considered that the immune system has been confronted with a certain viral sequence and mounted CD8 T cell responses specifically towards the infecting virus. One question is whether the immune system of HIV-infected individuals can create a new response towards a variant epitope in the case of vaccination. To study this in a comparable setting, we addressed the question how frequently a new CD8 T cell response can be generated after the occurrence of a viral escape mutation in its recognized epitope in a population not selected for a certain HLA allele.

## Methods

19 HIV-infected untreated subjects were sampled longitudinally (>6 months. We searched for CD8 T cell responses that declined over the course of untreated infection and sequenced the autologous virus of the early and late time point by RT-PCR. Recognition of wildtype and newly arising sequences was compared in peptide titration assays.

## Results

A total of 30 declining CD8 T cell responses were studied in detail and viral sequence analyses showed amino acid changes in 25 (83%) of these. Peptide titration assays revealed 12 (48%) viral escape mutations with 2 de-novo responses (17%). Here the de-novo response showed less effector functions than the original CD8 T cell response. In addition we identified 5 (20%) shifts in immunodominance. None of the subjects with adaptation to the changing virus carried the HLA alleles B27, B57 or B*5801.

## Conclusion

Our results show that CD8 T cell responses can adapt to the mutations of HIV. However it was limited to only 28% (7 out of 25) of cases in a cohort not expressing protective HLA alleles.

